# Role of Gubernaculum testis inervation during the process of testicular migration in human fetuses

**DOI:** 10.1590/S1677-5538.IBJU.2024.9914

**Published:** 2024-07-25

**Authors:** Luciano A. Favorito, Laura M. M. Favorito, Ana R. M. Morais, Francisco J. B. Sampaio

**Affiliations:** 1 Unidade de Pesquisa Urogenital Departamento de Anatomia da Universidade do Estado do Rio de Janeiro Rio de Janeiro RJ Brasil Unidade de Pesquisa Urogenital, Departamento de Anatomia da Universidade do Estado do Rio de Janeiro – UERJ, Rio de Janeiro, RJ, Brasil

**Keywords:** Gubernaculum, Testicular Diseases, calcitonin gene-related peptide

## Abstract

Purpose

The gubernaculum seems to be the most important anatomical structure in the testicular migration process. The objective of this paper is to review current literature regarding the role of gubernaculum testis nerves in testicular migration. We conducted a comprehensive literature review about the gubernaculum testis innervation. A PubMed database search was performed in April 2024, focusing on gubernaculum testis and cryptorchidism and genitofemoral nerve (GFN) and calcitonin gene-related peptide (CGRP) gene. The gubernaculum has its own nerve supply, the GFN, descending on the anteromedial surface of the psoas muscle from L1-L2 segments. The second phase of testicular descent is regulated by androgens and CGRP, released from the sensory nucleus of the GFN. The GFN doesn't directly play a role in testicular migration but there is a theory that shows a regulatory function of this nerve in hormonal action during this process. The gubernaculum testis has important structural alterations during the testicular migration and the genitofemoral nerve and CGRP gene are of great importance in this process. The genitofemoral nerve provides motor innervation to the cremaster muscle and gubernaculum, which helps regulate the position of the testes within the scrotum.

## INTRODUCTION

During the human fetal period the testes migrate from the abdomen to the scrotum traversing the abdominal wall and the inguinal canal between the 15th and the 28th week post-conception (WPC) ( 1 , 2 ). The most important factors involved in this process are: ( 1 ) intra-abdominal pressure ( 3 ); (b) development of the epididymis, spermatic vases and deferent ducts ( 4 ); (c) the genitofemoral nerve stimulus ( 5 ); (d) the hormonal stimulus ( 6 - 8 ); and (e) the gubernaculums development ( 9 , 10 ).

The gubernaculum seems to be the most important anatomical structure in the testicular migration process, by means of contraction and shortening, thus imposing traction strength on the testis and facilitates the transition of the testis through the inguinal canal ( 9 , 11 , 12 ).

One of the factors involved in cryptorchidism is the failure of the gubernaculum to migrate all the way to the scrotum ( 13 ). Structural studies conducted in patients with cryptorchidism reveal significant changes in the gubernaculum's structure, with a higher quantity of fibrous tissue and lower concentration of collagen than in the fetal gubernaculum ( 14 ). Studies about the gubernaculum nerves and their role in testicular migration are scarce in literature. The objective of this paper is to review the current literature regarding the role of gubernaculum testis nerves in testicular migration.

## MATERIAL AND METHODS

In this study we carried out a review about the role of the innervation of the gubernaculum testis in testicular migration and analyzed papers published in the past 50 years. A PubMed database search was conducted in April 2024 using the following Medical Subject Heading (MeSH) terms: "Testicular Migration" or ‘Gubernaculum Testis’ and either ‘Genitofemoral Nerve" or "CGRP gene" or ‘Undescended testis’ or ‘Cryptorchidism. Multiple free text searches were performed using the following terms individually through all fields of the records: ‘Genitofemoral Nerve" or "CGRP gene", ‘Undescended Testis’, and ‘gubernaculum testis’ or "Testicular Migration". In this review we found several papers in these databases, and we included only papers in English and excluded case reports, editorials and opinions of specialists.

## RESULTS

Testicular descent is regulated by hormonal and mechanic factors such the testosterone, insulin-like factor 3 (INSL3) and the gubernaculum ( 1 , 15 ). The gubernaculum is a ligament-like structure that guides the testes into the scrotum during fetal development ( 16 - 18 ). The process of testicular migration is very complex process and occurs in two distinct phases: Phase 1: Abdominal stage and Phase 2: Inguinal-scrotal stage ( 1 , 15 - 19 ). We show in this review some important aspects about the stages of testicular migration, the gubernaculum testis structure and innervation and the role of the genitofemoral nerve in this process.

### Abdominal Stage of testicular Migration

**Figure 1:**
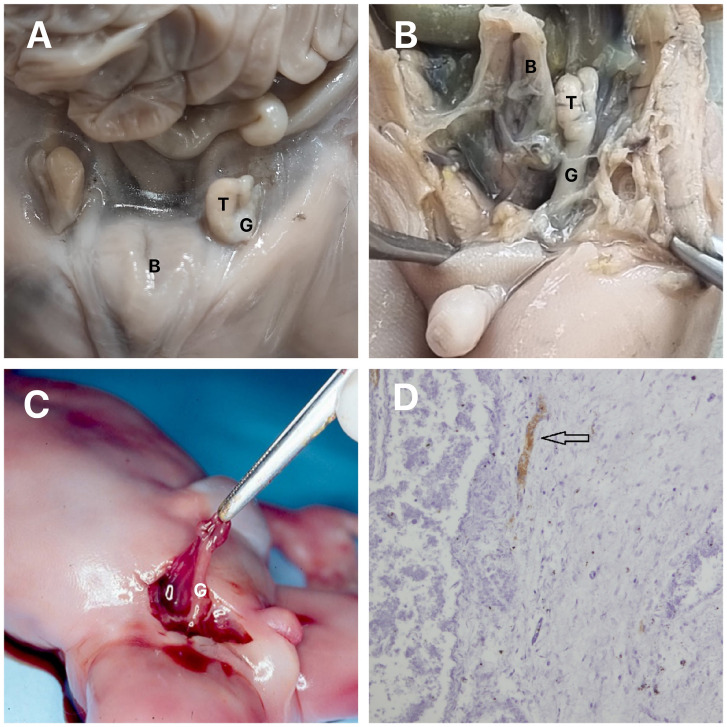
Gubernaculum Testis.

In this stage that begins around the 8th WPC and lasts until the 15th WPC the testis migrates from the abdomen to the internal inguinal ring when insulin-like hormone 3 (Insl3) from the Leydig cells stimulates the gubernaculum to swell, thereby anchoring the testis near the future inguinal canal as the fetus grows ( Figure-1 ) ( 20 ).

The figure shows some aspects of the gubernaculum testis during testicular migration. A) A fetus with 20 weeks post conception (WPC), the abdominal wall was opened and we can observe the testis in abdominal position (T) and the gubernaculum (G) near to internal inguinal ring, B= Bladder; B) In this figure we can observe a fetuses with 21WPC, the inguinal canal was opened to show the relationship of gubernaculum testis(G) with the inguinal canal, T=Testis, B=Bladder; C) The figure shows a fetuses with 24WPC with the testis in inguinal position, we can observe the dissection of the gubernaculum testis (G) and D) In this picture we can observe a photomicrography of the gubernaculum testis in a fetus with 20WPC in abdominal position. The arrow shows the gubernaculum innervation, Immunohistochemycal with tubuline antibody X400.

During the eighth week of gestation, the testis and mesonephros are linked to the posterior abdomen wall by a peritoneal fold ( 21 , 22 ). The portion of this fold called the diaphragmatic ligament degenerates, turning into the cranial portion of the gonadal mesentery. This structure is called the caudal gonadal ligament, which gives rise to the gubernaculum testis ( 1 , 22 ). One of the factors involved in cryptorchidism is the failure of the gubernaculum to migrate all the way to the scrotum ( 1 , 12 , 22 ). The influence of fetal androgens on the fetal gubernaculum's development is very important for the alterations of this structure, and the changes in its secretions can be one of the factors involved in cryptorchidism ( 1 ).

In the abdominal stage of testicular migration, the gubernaculum enlarges to hold the testes near the groin, regulated by INLS-3 ( 23 ). INSL-3 is secreted by the Leydig cells and controls gubernaculum swelling via its receptor, LGR8 (leucine-rich repeat containing G protein-coupled receptor 8, also known as GREAT or relaxin receptor 2), a process resulting in thickening of the gubernaculum because of increases in water, glycosaminoglycan and hyaluronic acid content ( 1 , 2 , 23 ). At this moment, the future inguinal canal is still only a space in the musculature of the anterior abdominal wall, where only mesenchyme tissue exists. In this region, the genital branch of the genitofemoral nerve crosses the abdominal wall and descends to the scrotum where it will innervate the cremaster muscle, and subsequently, in the caudal to cranial direction, will provide the nerve supply to the gubernaculum ( 1 , 19 ).

### Inguinal-Scrotal Stage of testicular Migration

The inguinal-scrotal stage is the transition of the testes through the inguinal canal until their definitive arrival in the scrotum that begins around the 20th WPC and lasts until the 30th WPC ( 1 , 23 ). During this stage, after the testis crosses the external inguinal ring the gubernaculum migrates across the pubic region to reach the scrotum under control of testosterone. The androgen acts indirectly via the GFN, which produces CGRP to control the direction of migration ( 24 ).

The passage of the testis through the inguinal canal occurs very quickly between 21 and 25 WPC ( 1 - 4 , 25 ). In a recent paper with more than 240 human male fetuses studied shows that all the fetuses older than 30 weeks already had the testes in the scrotum ( 25 ). Other authors, however, report that the testicular migration is only completed after the 32nd WPC ( 1 ). Heyns ( 1 ) found only 2.6% of the testes examined in his sample located in the inguinal canal, while Sampaio & Favorito ( 2 ), in a sample of 71 human fetuses, found 20.5% of the testes located there. Furthermore, 73.3% of these testes were in fetuses with ages between 21 and 25 WPC, indicating that in this period the migration through the inguinal canal intensifies. In the same study, all the fetuses older than 30 weeks already had the testes in the scrotum. Other authors, however, report that the testicular migration is only completed after the 32nd week post-conception ( 19 ).

The androgens stimulate growth and differentiation of the muscular part of the gubernaculum bulb, which facilitates the movement of the gubernaculum through the inguinal region by the traction resulting from this growth ( 12 , 26 ). Distally the gubernaculum approaches the inguinal region. At this moment, the future inguinal canal is still only a space in the musculature of the anterior abdominal wall, where only mesenchyme tissue exists. In this region, the genital branch of the GFN crosses the abdominal wall and descends to the scrotum where it will innervate the cremaster muscle, and subsequently, in the caudal to cranial direction, will provide the nerve supply to the gubernaculum ( 1 , 27 , 28 ).

### Gubernaculum Testis

The gubernaculum starts to develop in the human fetus during the sixth week of gestation, the same period when the germinative cells are arriving at the genital ridge ( 1 , 19 ).

In the eighth week of gestation, the testis and mesonephros are linked to the posterior abdomen wall by a peritoneal fold. As the mesonephros degenerates, the portion of this fold cranial to the testis, called the diaphragmatic ligament, also degenerates, turning into the cranial portion of the gonadal mesentery. This structure is called the caudal gonadal ligament, which gives rise to the gubernaculum testis ( 1 , 19 ).

At about the eighth week of gestation, a portion of the epithelium starts a small invagination from the coelomic cavity, across from the gubernaculum, slowly penetrating its mesenchymal substance. This invagination occurs bilaterally and is considered the start of the vaginal process. Some authors consider this phenomenon to be "active", involving the invasion of the gubernaculum by mesothelial cells ( 19 ), while others advocate that this phenomenon is "passive" and secondary to the increase in intra-abdominal pressure ( 1 , 19 ).

The growth of the vaginal process divides the gubernaculum into three parts: (a) the main gubernaculum, which corresponds to the portion covered by the visceral layer of the peritoneum of the vaginal process; (b) the vaginal gubernaculum, which corresponds to the portion that externally surrounds the parietal portion of the vaginal process, and (c) the infra-vaginal gubernaculum, corresponding to the caudal region of the gubernaculum, which has not been invaded by the vaginal process ( 19 ).

The maintenance of this undifferentiated mesenchyme along the inguinal canal and scrotum is essential for the downward extension of the vaginal process to occur, during which it follows the pathway created by dilation of the gubernaculum, forming the canal through which the testis will reach the scrotum ( 1 , 19 ).

The gubernaculum is a cylindrical structure, covered by a peritoneum on all sides except the posterior, where the testicular vessels and vas deferens are situated. Macroscopically, it looks like the Wharton's jelly of the umbilical cord ( 1 , 12 ). Histologically, it is composed of undifferentiated cells with elongated shape, surrounded by a large quantity of extracellular material, where it is impossible to identify smooth or striated muscle cells except in its distal end and in the peripheral portion ( 29 ) ( Figure-1 ).

The different parts of the gubernaculum undergo varied changes during testicular migration. The vaginal and infra-vaginal portions become proportionally longer as the testis starts to descend to the scrotum. At the same time, their diameter increases, a fact considered one of the most important mechanisms for dilating the inguinal canal to allow the testis to pass across the inguinal canal ( 1 ).

The gubernaculum's growth is divided into two phases, triggered by different hormonal stimuli ( 19 ). In the first, its volume increases and in the second it decreases in size, coinciding with the complete descent of the testis ( 1 ). The cremaster muscle presents structural alterations during this period as well ( 30 , 31 ). This muscle allows rhythmic contraction to guide the testis into the scrotum in rats and in humans, leading to eversion of the distal portion of the gubernaculum and contributing to its migration to the scrotum ( 30 ).

The first phase is marked by pronounced cell multiplication and accumulation of glycosaminoglycans, mainly hyaluronic acid. These substances act as hydrophilic agents and raise the quantity of water. There is also an increase in the amount of extracellular material, explaining the low cell density found at some points ( 1 ). The presence of myoblasts intensifies and there are changes in the number and arrangement of the collagen fibers and alterations of the elastic system.

In the second phase, the gubernaculum shrinks, particularly its length, normally accompanied by descent of the testis. This phenomenon appears to be androgen-dependent and brings substantial degradation of the glycosaminoglycans previously accumulated in the extracellular material, with consequent dehydration of this space and condensation of the gubernaculum ( 20 ). Although no estimates are available of the degree of shortening, some authors believe this acts together with other factors, causing the gubernaculum to convey the testis to the scrotum ( 1 , 19 ).

Understanding the relationship between regression of the gubernaculum and descent of the testis is vital to comprehension of how androgens control testicular migration. Studies have demonstrated an association between androgen deficiency, on the one hand, and failed regression of the gubernaculum and cryptorchidism on the other. In this situation, the gubernaculum appears to act as an obstacle to testicular descent ( 6 , 30 ).

The gubernaculum has its own nerve supply, the GFN, descending on the anterior and medial surface of the psoas muscle from L1-L2 segments ( 1 , 9 , 30 ). The 2nd phase of testicular descent is regulated by androgens and calcitonin gene-related peptide, released from the sensory nucleus of the GFN ( 5 , 15 ). In rodents, the active proliferation of the gubernacular tip and cremaster muscle, the muscle's rhythmic contraction, and the chemotactic gradient provided by the CGRP together result in migration of the testes into the scrotum. The importance of this mechanism is corroborated by experimental models where the sectioning of the genitofemoral nerve leads to cryptorchidism ( 12 , 23 ).

The gubernaculum's growth is divided into two phases, triggered by different hormonal stimuli ( 8 , 19 ). In the first, its volume increases and in the second it decreases in size, coinciding with the complete descent of the testis ( 28 ). The cremaster muscle presents structural alterations during this period as well ( 31 ). This muscle allows rhythmic contraction to guide the testis into the scrotum in rats and in humans, leading to eversion of the distal portion of the gubernaculum and contributing to its migration to the scrotum ( 31 ).

The first phase is marked by pronounced cell multiplication and accumulation of glycosaminoglycans, mainly hyaluronic acid. There is also an increase in the amount of extracellular material, explaining the low cell density found at some points ( 32 , 33 ). In the second phase, the gubernaculum shrinks, particularly its length, normally accompanied by descent of the testis. This phenomenon appears to be androgen-dependent and brings substantial degradation of the glycosaminoglycans previously accumulated in the extracellular material, with consequent dehydration of this space and condensation of the gubernaculum ( 29 ). Although no estimates are available of the degree of shortening, some authors believe this acts together with other factors, causing the gubernaculum to convey the testis to the scrotum ( 1 , 19 ). The connective tissue of the gubernaculum undergoes remodeling, so that at the end of migration it has essentially become a fibrous structure, rich in collagen and elastic tissue ( 29 ).

In a previous study with fetuses without anomalies and fetuses with Prune Belly syndrome the nerves of gubernaculum testis were analyzed ( 34 , 35 ). Prune belly syndrome (PBS) is a disorder characterized by deficiency or hypoplasia of the abdominal muscles and/or malformation of the urinary tract, such as large and hypotonic bladders, dilated and tortuous ureters and bilateral cryptorchidism ( 36 , 37 ). The main pathogenic theory of SPB is urethral obstruction that would cause distension of the urinary tract, preventing the normal development of the abdominal musculature and the descent of the testicles ( 36 ). Recently, important alterations in the gubernaculum testis structure were demonstrated in fetuses with PBS ( 20 ). Bilateral cryptorchidism is characteristic of prune belly syndrome ( 36 , 37 ). The contraction of the muscles of the abdominal wall, growth of the liver and intestines and accumulation of meconium all increase the pressure inside the fetal abdomen. According to several authors, this favors testicular migration ( 23 ). Contraction of the abdominal musculature is impaired in PBS. Mechanical obstruction due to bladder distention is another factor believed to hinder testicular migration in this syndrome ( 37 ). Another theory put forward to explain bilateral cryptorchidism in PBS is the structural alteration of the inguinal canal, which hampers the passage of the testis ( 37 ).

In this study about the gubernaculum in PBS with human fetuses ( 20 ), we observed a small quantity of nerves both in the gubernaculums of the control group and those of the PBS group (mean of 3.158%) without statistical differences ( Figure-1 ). This is the first study assessing and quantifying the distribution of the nerves of the human testicular gubernaculum. The small quantity of nerves presents in the gubernaculums studied could confirm the theory that the rhythmic contraction of the gubernaculum, mediated by stimulus from the genitofemoral nerve ( 7 , 8 , 20 , 35 ), has little importance in humans, but future researches will be necessary to clarify this topic.

The gubernaculums analyzed from the fetuses with PBS showed alterations in the concentrations of collagen and elastic fibers. We did not observe the processus vaginalis developing inside the gubernaculum in Prune Belly Syndrome. These structural alterations could be one of the factors involved in cryptorchidism in prune belly syndrome. The tissue changes in the gubernaculum testis during the fetal period suggest that it plays an active role in testicular migration.

### Genitofemoral Nerve

**Figure 2:**
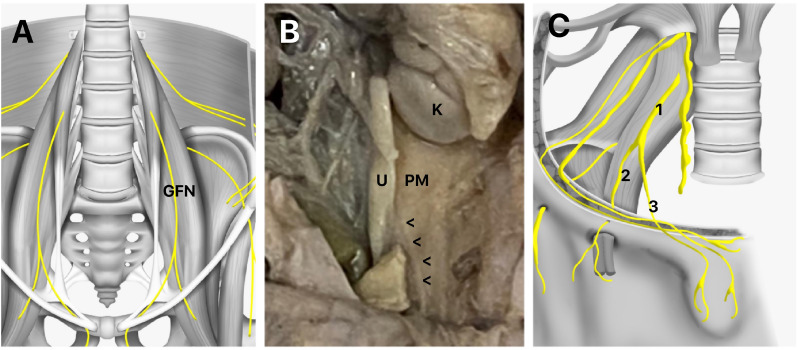
Genitofemoral Nerve Anatomy

The genitofemoral nerve is a mixed nerve that originates from the lumbar plexus, specifically from the ventral rami of the L1 and L2 spinal nerves. It has both sensory and motor components. Anatomically, the genitofemoral nerve divides into two branches: Genital branch and femoral branch ( 38 , 39 ) ( Figure-2 ).

A) The figure shows a schematic drawing of the genitofemoral nerve (GFN) and its relationship with the psoas major muscle; B) The figure shows a fetus with 22 WPC, the abdomen was dissected and we can observe the GFN (arrowheads) and the relationship of the nerve with the psoas major muscle (PM), U=Ureter, K=Left kidney; and C) The figure shows a schematic drawing of the genitofemoral nerve (1) ramification in femoral branch (2) and in the genital branch (3).

The genital branch supplies sensory innervation to the skin of the upper anterior thigh and the skin of the genital region, including the scrotum in males and the mons pubis and labia majora in females. In males, it also innervates the cremaster muscle, which is involved in the regulation of testicular position ( 38 , 39 ). The femoral branch innervates the femoral triangle, supplying sensory fibers to the skin over the femoral artery and vein, as well as the iliac lymph nodes. Overall, the GFN plays a crucial role in providing sensory innervation to the genital and upper thigh regions, as well as motor innervation to the cremaster muscle in males ( Figure-2 ) ( 38 , 39 ).

The GFN mainly provides sensory innervation to the genital region and motor innervation to the cremaster muscle, which helps regulate the position of the testes within the scrotum but doesn't directly influence their descent ( 1 , 5 ). The GFN doesn't directly play a role in testicular migration but there is a theory that shows a regulatory function of this nerve in hormonal action during this process ( 1 , 5 ).

**Figure 3:**
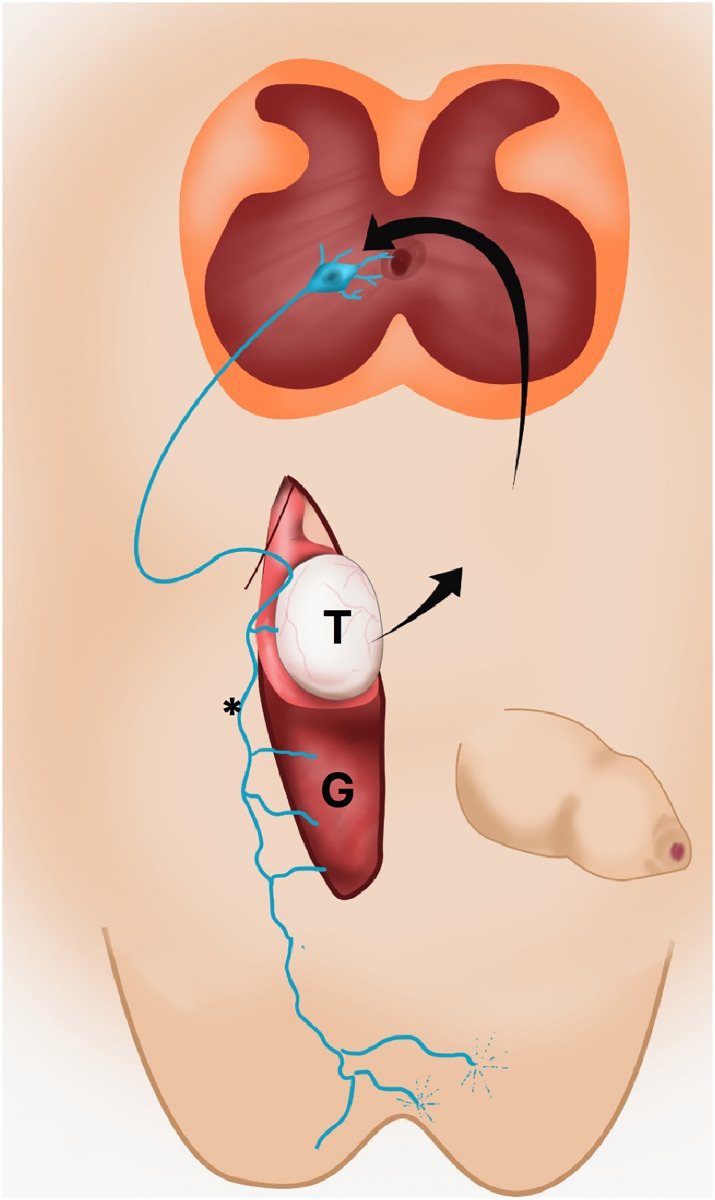
The figure shows a schematic drawing fetal of the genitofemoral nerve (*) action in testicular migration. The androgens originate in testis (T) masculinize the spinal nucleus of the genitofemoral nerve and result in an increase in the number of motoneurons in this region with consequent increase in secretion of the calcitonin gene-related peptide (CGRP) that induces structural modifications of gubernaculum testis (G) during testicular descent.

According to this theory, fetal androgens masculinize the spinal nucleus of the GFN and then the nerve itself ( 27 ). Testosterone appears to play an active role in testicular migration, inducing the development of important structures for testicular migration such as the vaginal process, the vas deferens, the epididymis, the inguinal canal and the scrotum. Another mechanism of action of testosterone would be through stimulation of the genitofemoral nerve, which would induce the production of calcitonin gene-related peptide (CGRP) that acts by stimulating the development of the testicular gubernaculum. This masculinization results in an increase in the number of motoneurons in this region with consequent increase in secretion of the calcitonin gene-related peptide (CGRP) ( Figure-3 ). This mechanism was studied in experimental models where the sectioning of the GFN leads to cryptorchidism ( 1 ). Increased CGRP levels lead to a rhythmic contraction of the testicular gubernaculum that would induce its migration to the scrotum ( 5 ).

Fetal and placental gonadotropins are also implicated in the process of testicular migration. These substances act by stimulating the production of testicular androgens, which induce the growth and development of the vas deferens, the epididymis, the vaginal process and the gubernaculum itself ( 8 ). It is well known that treatment of cryptorchidism with gonadotropins induces testicular migration at levels ranging from 25 to 55% of cases ( 28 ). Another endocrine substance involved in testicular migration would be descine ( 10 ). This androgen-independent secreted substance in the testis would play an important role in the growth of the gubernaculum mesenchymal cells. The gubernaculum would therefore be one of the fetal structures implicated in testicular migration, most modified by hormonal action ( 30 ).

The site of action of the CGRP is the neuromuscular junction. In experimental animals such as rodents, for example, there is an abundance of musculature, fortifying this hypothesis ( 5 ), but the human gubernaculum is basically composed of an abundant extracellular matrix with high concentrations of glycosaminoglycans ( 12 , 29 ), so this theory of CGRP-induced traction in humans is debatable. The indirect action of androgen via the GFN required for testicular descent may be one of the sites of anomalies in the putative multifactorial cause of cryptorchidism ( 40 ).

### Calcitonin Gene-Related Peptide Gene

The Calcitonin Gene-Related Peptide (CGRP) gene does not have a direct role in testicular migration. CGRP is primarily known for its involvement in vasodilation, neurotransmission, and pain modulation in the peripheral and central nervous systems. It is produced by sensory nerves and is involved in various physiological processes, including regulation of blood flow and inflammation. Testicular migration, on the other hand, is primarily regulated by hormonal factors, such as testosterone and INSL3, and the gubernaculum ( 1 , 9 ).

Insulin-like peptide 3 (INSL3) is a small peptide hormone of the insulin-relaxin family, which is produced and secreted by the fetal Leydig cells in the testes only following gonadal sex determination around 8WPC and represents a major secretory product uniquely from the male fetus ( 41 ). The INSL3 is very important during testicular migration because it influences gubernacular ligament thickening, anchoring the testis in the inguinal region and promoting the migration of the testis until the inner inguinal ring ( 41 ).

The integrity of the axis between the testis, hypothalamus and pituitary, which regulates testosterone production, is important for the testicular migration process. Cryptorchidism is a common event in pathologies on this axis, such as hypogonadotropic hypogonadism and 5-alpha reductase deficiency ( 15 , 23 ). Testosterone appears to play an active role in testicular migration, inducing the development of important structures for testicular migration such as the vaginal process, the vas deferens, the epididymis, the inguinal canal and the scrotum. Another mechanism of action of testosterone would be through stimulation of the genitofemoral nerve, which would induce the production of CGRP that acts by stimulating the development of the testicular gubernaculum.

The gubernaculum undergoes a "swelling reaction" during the transabdominal phase and is mainly under the control of INSL-3 and Mullerian Inhibitory Substance/Anti-Mullerian Hormone ( 1 , 9 ). The 2nd phase of testicular descent is regulated by androgens and calcitonin gene-related peptide (CGRP) release from the sensory nucleus of the GFN. In rodents, the active proliferation of the gubernacular tip and cremaster muscle, its rhythmic contraction, as well as the chemotactic gradient provided by the CGRP result in eventual migration of the testis into the scrotum. Cremaster muscle matures slower than other body muscles, and the persistence of immature myogenic proteins seen in cardiac muscle allows rhythmic contraction to guide the testis into the scrotum. Finally, remodelling of the cremaster muscle enables gubernacular eversion ( 1 , 42 , 43 ). Further understanding of the molecular regulators governing the structural and hormonal changes in the cremaster muscle may lead to new advances in the treatment of undescended testes.

While CGRP may indirectly influence some aspects of reproductive physiology, its role in testicular migration specifically is not well defined.

## CONCLUSIONS

The gubernaculum testis has important structural alterations during the testicular migration and the genitofemoral nerve and CGRP gene are of great importance in this process. In the first phase of testicular migration, the gubernaculum enlarges to hold the testis near the groin and in the second phase the gubernaculum migrates across the pubic region to reach the scrotum. The genitofemoral nerve provides motor innervation to the cremaster muscle and gubernaculum, which helps regulate the position of the testes within the scrotum.

## ABBREVIATIONS

GFN: = Genitofemoral nerve
CGRP: = calcitonin gene-related peptide
WPC: = week post-conception
INSL3: = insulin-like factor 3
